# Heart rhythm management optimisation of pacemaker recipients using remote monitoring: the HERO registry

**DOI:** 10.1007/s12471-025-01953-4

**Published:** 2025-05-09

**Authors:** Fleur W. Adriaansen, Jaap Seelig, Tim A. C. de Vries, Leonard Voorhout, Frank P. Brouwers, Balazs Manfai, Richard Derksen, Christiaan Aagenborg, Carine J. M. Doggen, Ron Pisters, Martin E. W. Hemels

**Affiliations:** 1 Department of Cardiology, Rijnstate Hospital, Arnhem, The Netherlands; 2 Radboud Health Academy, Radboud University Medical Centre, Radboud University, Nijmegen, The Netherlands; 3 Department of Cardiology, Radboud University Medical Centre, Radboud University, Nijmegen, The Netherlands; 4 Department of Clinical and Experimental Cardiology and Cardiothoracic Surgery, Amsterdam University Medical Centres, University of Amsterdam, Amsterdam, The Netherlands; 5 Amsterdam Cardiovascular Sciences, Heart Failure and Arrhythmias, Amsterdam, The Netherlands; 6 Department of Geriatric Medicine, Radboud University Medical Centre, Radboud University, Nijmegen, The Netherlands; 7 Clinical Research Centre, Rijnstate Hospital, Arnhem, The Netherlands; 8 Department of Health Technology and Services Research, Technical Medical Centre, University of Twente, Enschede, The Netherlands

**Keywords:** Pacemaker, Remote monitoring, Tachyarrhythmias, Atrial fibrillation, Atrial high-rate episodes

## Abstract

**Introduction:**

Current expert consensus recommends remote monitoring (RM) for cardiac implantable electronic devices (CIEDs). As this recommendation is primarily based on studies involving implantable cardioverter defibrillators (ICDs), the HERO (HEart Rhythm management Optimisation of pacemaker recipients using remote monitoring) study aimed to reinforce this recommendation for pacemaker recipients.

**Methods:**

The exploratory, retrospective, single-centre HERO study included 203 patients with an increased stroke risk (CHA_2_DS_2_-VASc score ≥ 2) but without a history of atrial fibrillation or flutter, who received a pacemaker between January 2016 and April 2018. Occurrence and detection time of atrial and ventricular arrhythmias were analysed in patients with RM (RM+; *n* = 60) and those without RM (RM−; *n* = 143), together with CIED adverse events, cardiology visits and anticoagulation adjustments.

**Results:**

The median age of the patients was 80 (73–85) years, with 55.2% being men. During a median follow-up of 5.0 years, 53.7% were diagnosed with at least one arrhythmic event (RM+ 60.0% vs RM− 51.0%, *p* = 0.28). The median time from pacemaker implantation to detection of first arrhythmic event was 2.5 (0.5–8.2) years in the RM+ group versus 2.8 (1.2–8.0) years in the RM− group (hazard ratio 0.89; 95% confidence interval 0.59–1.35; *p* = 0.58). There were no differences in the number of adverse events or anticoagulation adjustments during follow-up. More CIED telephone consultations were conducted in the RM+ group.

**Conclusion:**

A substantial proportion of pacemaker patients experienced one or more arrhythmic events during follow-up. The HERO study did not demonstrate a difference in time to detection of the first event when using remote monitoring.

## What’s new?

- The HERO study is the first to study the efficacy of remote monitoring (RM) in pacemaker recipients in a Dutch population.
- The HERO study shows that the incidence of (short) arrhythmic events in pacemaker recipients with an increased stroke risk and no history of atrial fibrillation or flutter is high; 54% experienced one or more events during follow-up.
- The HERO study shows that RM has a very high acceptance (95%) and minimal dropout rate (7%). Only 1 out of 60 patients had to stop using RM due to a technical malfunction. In combination with other studies showing that it is safe to reduce the follow-up frequency of patients using RM, these results could mean a significant reduction in the healthcare burden.

## Introduction

Remote monitoring (RM) of cardiovascular implantable electronic devices (CIEDs) enables the early detection of (sub)clinical events, such as arrhythmias and technical malfunctions. RM has been shown to be effective [1] and safe, allowing for a reduction in the frequency of follow-up visits for patients using RM [2, 3]. Recent expert consensus assigns a class I recommendation for the use of RM as the standard of care in all CIED patients [4]. However, RM has not yet been fully adopted in many European countries, with reimbursement policies posing a significant barrier to full implementation [5].

The implementation of RM as the standard of care for pacemaker recipients in particular is lagging behind. This is likely because the recommendation in the expert consensus document is primarily based on studies involving implantable cardioverter defibrillators (ICDs) and cardiac resynchronisation therapy defibrillators. In the Netherlands, RM is the standard of care only for ICD patients, but not for pacemaker recipients, despite increasing evidence that RM allows for more rapid detection of adverse events compared to conventional monitoring in pacemaker patients [6, 7].

Few prior studies have investigated the time to detection of arrhythmic events in pacemaker patients using RM, with most focusing on atrial arrhythmias, specifically atrial high-rate episodes (AHREs) [6, 7]. AHREs are asymptomatic supraventricular tachycardias detected by a CIED [8] and are associated with an increased thromboembolic risk, although this association is weaker compared to clinical atrial fibrillation (AF) [9, 10].

**Fig. 1:**
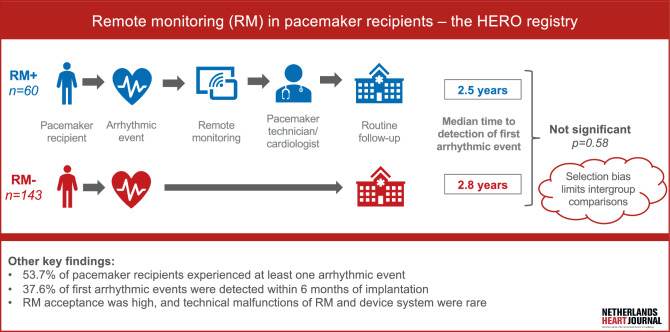
Infographic: Remote monitoring in pacemaker patients—the HERO registry. *RM+* patients with remote monitoring, *RM−* patients without remote monitoring

The primary aim of the exploratory HERO registry is to investigate whether arrhythmias—a composite of AHREs, clinical atrial arrhythmias, and ventricular arrhythmias—are detected earlier using RM in a Dutch pacemaker population. Additionally, the time to detection of device- or lead-related adverse events, the number of hospital visits, and changes in anticoagulation prescriptions between patients using RM and those without RM will be assessed (Infographic: Fig. 1).

## Methods

### Study design

The HERO (HEart Rhythm management Optimisation of pacemaker recipients using remote monitoring) registry is an exploratory, single-centre, retrospective cohort study conducted at Rijnstate Hospital in Arnhem, the Netherlands. The study was approved by the local ethics committee and conducted in accordance with the Declaration of Helsinki and European laws on patient rights and data protection.

### Study setting and patients

Patients ≥ 18 years with an increased stroke risk (i.e. CHA_2_DS_2_-VASc score ≥ 2) who received a pacemaker with both an atrial and ventricular lead were included in the HERO registry. Patients with a history of AF or atrial flutter (AFl) were excluded.

At Rijnstate Hospital, it is standard practice for pacemaker recipients to receive either a Biotronik (Berlin, Germany) or Boston Scientific (Marlborough, MA, USA) pacemaker. Between 1 January 2016 and 30 April 2018, patients receiving a Biotronik pacemaker were offered Biotronik’s RM device as part of this exploratory study. All eligible patients who received a pacemaker within this period were included, and those using remote monitoring (RM+) were compared with those not using remote monitoring (RM−).

Medical records of eligible patients were retrospectively assessed. The study follow-up period for each participant extended from the date of pacemaker implantation to the last follow-up date, ending on 7 August 2024. Patients were considered lost to follow-up if they were deceased, had discontinued follow-up at Rijnstate Hospital, had switched to AAI or VVI pacing mode, or had received a different device type or model.

### Study objectives

The primary objective was to assess the difference in time to detection of the first arrhythmic event between the RM+ and RM− groups. The endpoint was measured as the time from pacemaker implantation to the first arrhythmic event, including all atrial and ventricular arrhythmias.

Secondary endpoints included the time to detection of the first device- or lead-related adverse events, the numbers of outpatient clinic visits and hospital admissions for all cardiac causes, and the number of patients for whom anticoagulation (including antiplatelet therapy) prescription was adjusted during follow-up. All changes in anticoagulation or antiplatelet therapy were included, not only changes in the context of arrhythmias.

### Remote monitoring

Biotronik’s CardioMessenger device automatically gathers pacemaker data daily, provided the pacemaker is within range. All transmitted RM data can be reviewed by a physician via the Home Monitoring system on a secure website. When a (programmed) abnormality is detected, an alert is sent to the hospital, where it is reviewed daily by a pacemaker technician.

Alerts for arrhythmias were activated for all RM+ patients. When an atrial event exceeded 6 min, patients were contacted and asked whether they were experiencing symptoms. In cases of AHRE > 24 h or symptomatic atrial arrhythmias, anticoagulation was initiated in accordance with guidelines. Patients with subclinical AF were informed and invited to participate in either the ARTESIA or NOAH-AFNET 6 trial. Regarding ventricular arrhythmias, action was taken in cases of sustained ventricular tachycardia episodes (≥ 30 s) or recurrent non-sustained ventricular tachycardia.

### Follow-up

As this is a retrospective study, all patients received standard care. Outpatient clinic visits were scheduled for 2 weeks and 2 months after implantation, followed by annual pacemaker readouts for both groups. Additional readouts were conducted if clinically indicated. Biotronik pacemaker recipients were offered the CardioMessenger device either at the 2‑week post-implantation visit or during the annual readout.

### Statistical analysis

Continuous data were expressed as means ± standard deviation or medians (interquartile range), while categorical data were displayed as counts and percentages. Continuous variables were compared using an independent sample *t*-test or Mann-Whitney U test, as appropriate, and categorical variables were compared using Fisher’s exact test.

Cumulative incidence curves created by Kaplan-Meier analysis were used to visualise the time to the primary endpoint as well as time to the secondary endpoint, i.e. first lead- or device-related adverse event. Hazard ratios (HRs) with 95% confidence intervals (CIs) for the time differences between the two groups were calculated using a Cox proportional hazards model, adjusting for confounders if necessary.

The number of outpatient clinic visits and hospital admissions for all cardiac causes was calculated as events per patient-year per group. The proportions of patients with changes in anticoagulation prescriptions during follow-up were compared using Fisher’s exact test. Statistical analyses were performed using SPSS version 29.0.1.0 (IBM Corp., Armonk, NY, USA).

## Results

### Patients and follow-up

A total of 203 patients (median age 80 (73–85) years, 55.2% men) were included in the HERO registry: 60 (29.6%) in the RM+ group, and 143 (70.4%) in the RM− group. In the RM− group, 86 patients (60.1%) received a Biotronik pacemaker, while 57 (39.9%) received a Boston Scientific pacemaker. The baseline characteristics are displayed in Tab. 1. Of the Boston Scientific pacemaker recipients, 33.3% had a medical history of heart failure. The mean CHA2DS2-VASc score was 3.6 ± 1.3 in the RM+ group and 3.6 ± 1.4 in the RM− group (*p* *=* 0.96).

**Table 1:** Baseline characteristics of the study groups

Characteristics	RM+ (*n* = 60)	RM− (*n* = 143)	*p*-value
Age, years	78.5 (73.0–84.8)	80.0 (73.0–87.0)	0.19
BMI, kg/m^2^	26.0 (23.4–28.9)	25.6 (23.1–28.7)	0.68
*Type of intervention*			
Implantation	58 (96.7%)	102 (71.3%)	< 0.001
Replacement	2 (3.3%)	41 (28.7%)	
*Cardiac pacing indication*			
Sinus node dysfunction	10 (16.7%)	21 (14.7%)	0.83
AV block	45 (75.0%)	95 (66.4%)	0.25
CRT	1 (1.7%)	17 (11.9%)	0.03
Others	4 (6.7%)	7 (4.9%)	
Unknown		3 (2.1%)	
*CHA*_*2*_*DS*_*2*_*-VASc risk factors*			
Congestive heart failure	5 (8.3%)	29 (20.3%)	0.04
Hypertension	37 (61.7%)	54 (37.8%)	0.002
Age ≥ 75 years	41 (68.3%)	102 (71.3%)	0.74
Age 65–74 years	17 (28.3%)	27 (18.9%)	0.14
Diabetes mellitus	13 (21.7%)	31 (21.7%)	1.0
Stroke/TIA/TE	8 (13.3%)	28 (19.6%)	0.32
Vascular disease	15 (25.0%)	49 (34.3%)	0.25
Female gender	30 (50.0%)	61 (42.7%)	0.36
*Comorbidities*			
Cardiomyopathy	4 (6.7%)	12 (8.4%)	0.78
COPD	3 (5.0%)	22 (15.4%)	0.06
Sleep apnoea	1 (1.7%)	5 (3.5%)	0.67
Kidney failure	10 (16.7%)	18 (12.6%)	0.50
*Therapy*			
Beta blocker	19 (31.7%)	48 (33.6%)	0.87
ACE inhibitor	23 (38.3%)	44 (30.8%)	0.33
ARB	11 (18.3%)	35 (24.5%)	0.37
MRA	3 (5.0%)	12 (8.4%)	0.56
Statin	26 (43.3%)	64 (44.8%)	0.88
Antiarrhythmic^a^	2 (3.3%)	3 (2.1%)	0.63
Anticoagulation	1 (1.7%)	21 (14.7%)	0.005
Antiplatelet	30 (50.0%)	78 (54.5%)	0.64

*ACE* angiotensin converting enzyme, *ARB* angiotensin receptor blocker, *AV* atrioventricular, *BMI* body mass index, *COPD* chronic obstructive pulmonary disease, *CRT* cardiac resynchronisation therapy, *MRA* mineralocorticoid receptor antagonist, *RM+* patients with remote monitoring, *RM−* patients without remote monitoring, *TE* thromboembolism, *TIA* transient ischaemic attack

^a^Antiarrhythmics class I or III according to the Vaughan Williams classification

Among the Biotronik pacemaker recipients without RM, the vast majority (96.5%) were never offered the CardioMessenger system, and the remaining patients (*n* = 3) had either declined or had passed away before receiving the RM system. In the RM+ group, the median time from pacemaker implantation to acceptance of RM was 76.0 (61.0–194.0) days. Four RM− patients stopped using RM during follow-up.

**Fig. 2:**
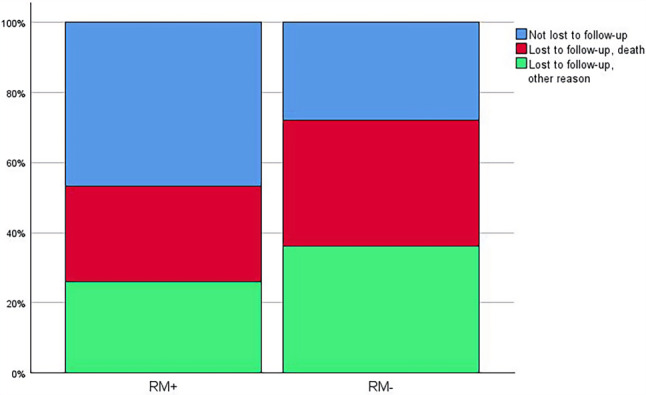
Follow-up status (in percentages of total group) per study group. *RM+* patients with remote monitoring, *RM−* patients without remote monitoring

The median follow-up was 5.8 (3.7–6.7) years in the RM+ group, and 4.4 (1.9–6.3) years in the RM− group (*p* *=* 0.004). The follow-up percentages for the different study groups are shown in Fig. 2.

### Incidence of arrhythmic events

In 109 patients (53.7% of the study population), at least one arrhythmic event was detected during follow-up. Of these, 98 (89.9%) had a device-detected event as the first event, and in 78 (71.6%) no clinical arrhythmia was reported. Furthermore, 79.8% of the first events were atrial tachyarrhythmias, while 22 patients (20.2%) experienced a ventricular first event. In 41 patients (37.6%), the first event was detected within 6 months of implantation.

In the RM+ group, at least one arrhythmic event occurred in 60.0% of the patients during follow-up, compared to 51.0% in the RM− group (*p* *=* 0.28). When comparing Biotronik pacemaker recipients with Boston Scientific pacemaker recipients, significantly more Boston Scientific patients were diagnosed with at least one arrhythmic event (47.9% vs 68.4%; *p* *=* 0.01). Although insufficiently reported, the duration of device-detected events is shown in Tab. 2.

**Table 2:** Duration of device-detected atrial events, divided into events lasting up to 6 min and those longer than 6 min. The reporting of event duration was incomplete, particularly in the RM− group. Patients whose first event was ventricular (*n* = 22) and patients who experienced a clinical arrhythmia without a device-detected event being reported (*n* = 2) are not included in this table

	RM+ (*n* = 60)	RM− (*n* = 143)
Number of patients with at least one arrhythmic event	36 (60.0%)	73 (51.0%)
Number of patients with a first device-detected atrial arrhythmia	29 (48.3%)	56 (39.2%)
– Duration of first device-detected event not reported	7 (24.1%)	25 (44.6%)
– First device-detected atrial event $\leq$ 6 min	9 (31.0%)	16 (28.6%)
– First device-detected atrial event $>$ 6 min	13 (44.8%)	15 (26.8%)

*RM+* patients with remote monitoring, *RM−* patients without remote monitoring

### Detection of arrhythmic events

**Fig. 3:**
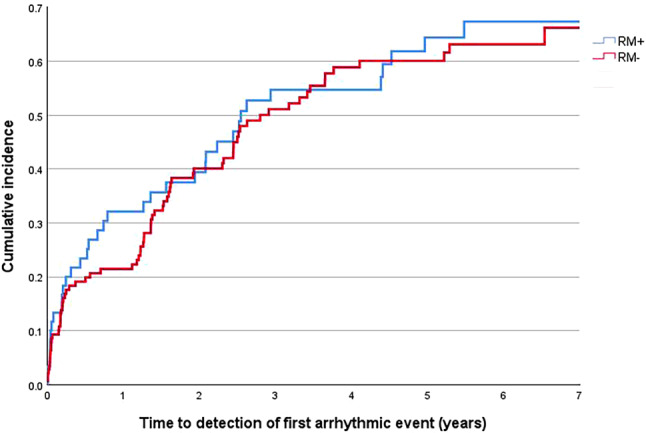
Cumulative incidence curves created by Kaplan-Meier analysis of time from implantation to detection of first arrhythmic event per study group. *RM+* patients with remote monitoring, *RM−* patients without remote monitoring

The median time between pacemaker implantation and detection of the first arrhythmic event was 2.5 (0.5–8.2) years in the RM+ group and 2.8 (1.2–8.0) years in the RM− group (HR = 0.89; 95% CI 0.59–1.35; *p* *=* 0.58), adjusting for heart failure and hypertension. The cumulative probability of an individual being diagnosed with an arrhythmic event at any time after implantation is shown in Fig. 3.

Also after correction for duration of the first event (6 min or less vs more than 6 min), no significant difference between RM+ and RM− was demonstrated (HR = 0.86; 95% CI 0.55–1.34; *p* *=* 0.49).

### Secondary objectives

Sixteen patients (7.9%) experienced one or more lead- or device-related adverse events, most occurring shortly (median time to first event: 11 (4–300) days) after pacemaker implantation. Adverse event rates were 6.7% in the RM+ group and 8.4% in the RM− group (*p* *=* 0.78). There were not enough events to conduct a reliable survival analysis.

The numbers of outpatient clinic visits and hospital admissions are shown in Tab. 3. In the RM+ group, evidently more CIED-related telephone consultations were conducted. Differences in CIED outpatient clinic visits and hospital admissions between the groups were negligible.

**Table 3:** Numbers of all cardiac outpatient clinic visits, cardiac implantable electronic device (CIED) outpatient clinic visits, all cardiac telephone consultations, CIED telephone consultations and hospital admissions for all cardiac causes

	RM+		RM−	
	Total no. of events	Events/patient-year	Total no. of events	Events/patient-year
Outpatient clinic visits for all cardiac causes	443	1.43	1060	1.81
– Pacemaker visits	326	1.05	648	1.11
Consultations by telephone for all cardiac causes	87	0.28	280	0.48
– Pacemaker consultations	36	0.12	23	0.04
Hospital admissions	14	0.05	42	0.07

*RM+* patients with remote monitoring, *RM−* patients without remote monitoring

The proportion of patients undergoing an adjustment in anticoagulation or antiplatelet prescription during follow-up was 38.3% in the RM+ group compared to 31.5% in the RM− group (*p* *=* 0.42).

## Discussion

The HERO retrospective single-centre pilot study did not demonstrate a significant difference in time to detection of the first arrhythmic event between pacemaker recipients with and without RM. The HERO registry is a valuable exploratory study with a long follow-up period, up to 8.2 years, and a median follow-up of 5.0 years. It demonstrated that a substantial proportion of pacemaker recipients (53.7%) experienced one or more arrhythmic events. This estimate may even be conservative, given that clinically non-relevant events were not consistently documented in the health records system.

The SETAM study, a French multicentre randomised trial, also including only pacemaker patients, found that the time between pacemaker implantation and the first treated atrial tachyarrhythmia was 110 days shorter in the RM group [7]. Similarly, the multicentre, prospective, non-randomised RAPID study demonstrated an AHRE evaluation delay of 2 versus 81 days between the RM and non-RM groups, both of which consisted solely of pacemaker recipients [6]. In the HERO study, the Kaplan-Meier curves indicated a noticeably higher event detection rate in the RM+ group during the first 1.5 years after implantation. Although the overall difference between the groups was not statistically significant, this early post-implantation difference aligns with the findings of the SETAM and RAPID studies and may be of clinical relevance.

Of the first arrhythmic events, 37.6% were detected within 6 months of implantation—a period during which more frequent outpatient clinic visits are typically scheduled in the current healthcare system. This reduces the potential time gain in event detection when using RM, especially since the median time from implantation to RM acceptance was 76 days. Previous studies have shown that it is safe to reduce the frequency of visits for patients using RM, extending the follow-up interval to up to 2 years [2, 3]. The HERO registry showed an increase in CIED-related telephone consultations in the RM+ group, without a significant reduction in outpatient clinic visits. This increase can probably be explained by the need to call patients after detection of (clinically relevant) events on RM, which has not yet been offset by fewer outpatient visits. The logistics of managing this new patient group, including determining follow-up frequency, the division of tasks between pacemaker technicians and cardiologists, and the documentation of device-detected events in the health records system, remains a relevant topic.

Most first arrhythmic events appeared to be tachyarrhythmias. Another topic of debate is the clinical implications of these AHREs, also referred to as subclinical AF, as two major trials on anticoagulation in patients with subclinical AF presented conflicting results [11, 12]. The ECS guidelines for AF give a class IIb recommendation for initiation of anticoagulation in patients with asymptomatic device-detected AF and elevated thromboembolic risk [13]. The HERO registry did not show significant differences in changes in anticoagulation or antiplatelet prescription for any cause, although between-group differences limit the interpretability of these results.

### Strengths and limitations

The exploratory HERO registry provides real-world data from consecutive patients receiving a pacemaker between January 2016 and April 2018, with an extended follow-up period. RM acceptance was high, with only three patients either declining RM or passing away before receiving the system, while 60 patients accepted it. Nearly all patients in the RM+ group used RM throughout the entire follow-up, with a minimal number of technical malfunctions. This high acceptance and ease of use, combined with the demonstrated safety of reducing the frequency of follow-up visits, could significantly alleviate the increasing burden on healthcare systems.

An important limitation of the HERO study is the moderate comparability between the study groups. A selection bias was observed, as patients using anticoagulation before pacemaker implantation were rarely offered RM due to the minimal clinical benefit of detecting early atrial arrhythmia in these individuals. Additionally, the RM+ group included more patients with hypertension, whereas the RM− group had a higher prevalence of heart failure and cardiac resynchronisation therapy pacemakers, largely driven by the higher proportion of heart failure patients receiving Boston Scientific pacemakers. Consequently, drawing definitive conclusions from group comparisons is challenging. Nevertheless, the descriptive data of the study population, such as the high incidence of device-detected arrhythmias, likely reflect the real-world pacemaker population. Notably, fewer patients in the RM+ group were lost to follow-up, which might indicate that this group represents a healthier population. However, the high incidence of arrhythmias even in these potentially healthier RM recipients highlights the potential benefit of RM.

Since all Boston Scientific pacemaker recipients were in the RM− group, the time-to-event analysis may have been influenced by the significantly higher number of first events detected in patients with a Boston Scientific pacemaker. Heart failure is seen as cause and consequence of both AF and ventricular arrhythmias [14], making events more likely in a group with more patients experiencing or having experienced heart failure symptoms. Additionally, Boston Scientific software classifies ventricular arrhythmias slightly differently than Biotronik software (4 versus 8 consecutive ventricular beats, respectively), possibly leading to the detection of more events than those classified by Biotronik software. However, to maintain statistical power, Boston Scientific pacemaker recipients were not excluded from this exploratory study.

## Conclusion

While the HERO study demonstrated a high incidence of arrhythmic events in pacemaker recipients with an increased stroke risk and no history of AF or AFl, it did not show a difference between patients with and without RM in the time to detection of the first arrhythmic event. However, as this study is limited by its retrospective design, a prospective, preferably randomised, trial is needed. Although formally not an endpoint, the HERO study demonstrated high acceptance and ease of use of RM, suggesting that, when used efficiently, RM could significantly reduce the healthcare burden.
